# Hospital Administration.—Alcohol and Milk Consumption

**Published:** 1887-09-17

**Authors:** 


					Sept. 17, 1887.] THE HOSPITAL. 4i3_
Hospital Adjyunstration ? Alcohol and Milk Consumption.
r
Value Of the Clonsunlption of Milk and Alcohol at nine General and thirteen Spebial Hospitals,
during the years 1875 and 1885 respectively.
Name ofr Hospital.
German Hospital ..
King's College Hospital ..
London Hospital
Metropolitan Free Hospital
Royal Free Hospital
St. George's Hospital
Training Hospital, Tottenham
University College ..
West London
9 General Hospitals ..
CityofLond. Hosp. forDis.ofChes
Brompton Consumption Hospital
Royal Hosp. for Dis. of the Chest
East London Hosp. for Children
Hospital for Sick Children
Grosvenor Hp. forWomn. & Chldrn
Female Lock Hospital
Central Lond. Ophthalmic Hospita
Royal Lond. Ophthalmic Hospita
City Orthopaedic Hospital
Royal Orthopaedic Hospital
St. John's Hosp. for Dis. of the Ski;
London Homceopathic Hospital .
13 Special Hospitals ..
Grand Total ..
Total Coil
sumption
of Milk.
Wine and
Spirits.
? s. d.
299 1 6
305 7
913 4
39 16 6
372 " 4
Si7 4 ?
67 2 3
327 " 4
4615 11 10
2841 18 6
266 1 11
655 o o
65 7 10
146 o o
134 15 7
28 16 3
no 18 o
4 7 4
III o o
35 o o
87 10 0
1 16 8
209 511
234 14 11
774 o o
49 16 3
3200
93 9 10
10 15 o
59 13 2
19c
72 17 4
34 3 4
1855 19
1383 3 10
6471 .11 44225 2 4
1875.
Malt Liquors.
Patients.
? s. d.
97 7 8
34 i4 6
330 18 io
3 7 6
466 8 6
72 6 7
128 o o
19 o o
79 17 10
9 10 o
60 12 10
161 10 5
700
30 15 6
568 13 2
1035 1 8
? s. d.
6140
160 19 11
905 16 3
124 13 o
138 10 9
230 2 8
19 12 o
250 12 1
171 o o
2062 10 8
36 3 3
95 o o
44 17 ?
890
046
80 12 9
54 6 6
319 13 o
2382 3 8
Spirits of
Wine.
Total Con-
sumption
of Alcohol.
? s. d. ? s. d.
67 11 o 525 4 2
102 15 4 569 2 9
417 7 6 2236 7 10
17 14 o 182 3 6
103 1 8 648 18 3
172 10 611250 16 o
284 92 10 1
169 18 o 748 1 s
86 o o 257 o o
1139 6 4 6510 4 o
408 4 9
1293 o o
103 10 3
61 o 0
272 10 4
35 18 o
125 6 o
1 13 6
240 15 3
840
133 3 3
1 15 o
93 3 8
2778 4 o
9288 8 o
65 o
296 o
8 17
10 o
99 2
7 4
5 o
6 '7 6
1 4
I 10
1 IS
4 13
506 14 o
1646 O 4
No. of In-]
Patients,
rS7F-7-
1235
'777
63?3
158
1625
3820
342
2007
556
1782s
819
927
145
400
1273
45
72
1294
93
5
461
5534
23357
Total Con- wi d
HK" Spirits.
6608 i 10
449 " 5
iii8 o o
55 6 o
338 o o
416 19 3
50 4 1
195 11 10
28 8 7
128 17 o
41 o o
75 7 2
73 9 9
358 9 2
3329 4 3
9937 6 1
? s. d.
201 2 6
231 17 o
S25 4 7
7 12 4
267 15 o
481 17 7
183 12 o
110 o o
2009 I o
48 14 II
8 11 8
46 12 1
1 1 4
43 5 4
10 16 o
52 0 I
45 11 3
939 1 11
2948 2 11
1885.
Malt Liquors.
Staff.
Patients.
55 16 i
300 9 11
? s.
48 17
19 3
349 2
16 2
70 2
140 6
213 3
926 16 9
954 18 1
71 8
73 o
7 IS 6
108 o
38 14
27 7 o
38 17 I
32
123
3 14
5? 8
2 6 10
167 15
16 o
46 8
6 4
441 14 7
1396 12 8
Spirits
of Wine.
? d.
go 3 6
180 2
767 3
9 7
70 10
164 4 3
2 8 4
265 2 6
50 o
397 o
29 r2
34 o
58 5
10 17 10
6 6
11 9
1 3
5
Total Con-
sumption
of Alcohol.
? s. d.
458 8 5
493 18 6
2031 o 2
33 2 6
464 3 8
1096 18 4
284
661 18 1
258 ? 0
5489 17 10
299 19 5
1073 o o
49 9 o
220 o o
145 14 5
23 3 6
103 6 1
14 12 3 |
222 9 6
11 3 o
56 8 o
94 i7 2
100 19 6
2415 1 10
7904 19 8
No. of In"|
Patients.
1387
2011
8106
285
1728
3669
502
2860
1197
21745
989!
16461
122'
978
1394
93
133
2118
109
143
*34
656
8515
30260
1875 and 1885 Compared.
Percentage ofincrease(+)
or decrease (?)
in No.
of In-
patients.
in consumption of
(a) | (J)
Milk. Alcohol
+ 12.3
+ 13-2
+ 28.8
+ 8o-3
+ 6.3
? 39-5
+46.8
+42-5
+ U5-3
+22.0
+ 20.7
+77-6
?iS-9
+ 144-5
+95-I
+ 106.7
+84.7
+63.7
+ 53-7
+42.3
+ S3.9
+ 29.1
+ 66.5
+ 70.2
+ 47-2
-64.1
+ 23.3
+11.9
+ 104.5
+ 38-9
+ 187.7
+43-2
+ 68.8
+70-7
-I5.4
+ I3I-5
+ 208.9
+ 72.4
+ 76.6
+ 552-9
+ 16.1
+ 17.1
-13-8
+ 7I-3
+79-4
+ 53-6
-12-8
-13.2
-9.2
-81.9
-28-5
-12.3
- 96.8
-11.5
+ -4
-15-7
-26.5
-17.0
-51.9
+260.7
-465
-36.1
-17.6
+781-8
-7-5
+ 36.0
-57-9
' +8*3
-J3-i
-14.9
We to-day publish, some further tables
, j and statistics concerning:
An Appeal to , , .
Hospital nine general and thirteen
Secretaries, special metropolitan hospi-
tals, in continuation of the papers on the
Consumption of Alcohol, which appeared
on pp. 5, 41, and 75 of Volume I. It may-
be observed that as we have now, at
much labour and expense, completed the
figures for the year 1885, it would be
very helpful if the secretaries of the
various institutions would in future
arrange that the accounts published in
the annual reports show the value of (a)
the wine and spirits, (6) the spirits of
wine, and (c) malt liquors (1) used by
staff, (2) by patients, consumed each
year. If this was done, no returns would
be necessary, as all the figures could then
be extracted without difficulty. We may
here observe that we are accumulating
much information which cannot fail to
be useful to hospital committees, secre-
taries, and officials, and that anyone who
will write to us when in a difficulty upon
any point shall gladly be helped to the
fullest extent in our power.
Our attention has been called to the
_ . .. .TTT. fact that the omission of
Spirits of Wine. spiritg of wine from the
tables previously given has lessened their
value to a considerable extent. It will be
seen on reference to the return pub-
lished to-day that the value of spirits
of wine consumed in the nine general
and thirteen special hospitals included
amounted to ?1,646 in 1875, and to ?2,247
in 1885, an increase of rather more than
?600, or 36.4 per cent. This fact shows
the importance of including the expendi-
ture on spirits of wine under a separate
heading in the accounts, seeing that the
increase in the number of in-patients
during the same period was only 29.1 per
cent., or 7.3 per cent, less than the in-
crease on this item alone.
We have not space to apply the figures
in detail to-day, but the
table is so clear as to enable
Remarks. , , ,
anyone to do this without
difficulty. The total result shows that
whereas the consumption of milk has in-
creased in the case of the nine general
hospitals by 42.2 per cent., the increase
in the case of the thirteen special hos-
pitals amounts to no less than 79.4 per
cent. On the other hand the consump-
tion of alcohol at the nine general hos-
pitals has decreased to the extent of 15.7
per cent., the only exception to this being
the West London, where there has been
an increase in alcohol equal to .4 per
cent. Taking all the thirteen special
hospitals, the total decrease amounts to
13.1 per cent., although there has been
an increase in the number of patients of
53.9 per cent. There has been a consider-
able increase in the consumption of alco-
hol at the East London Hospital for
Children, where the number of in-
patients was more than doubled in the
ten years. Other increases are manifest,
and these tables will give the initiated
an insight into the relative efficiency
and management of each institution,
besides being helpful in other respects.

				

